# Using advanced oxidation protein products and ischaemia-modified albumin to monitor oxidative stress levels in patients with drug-induced liver injury

**DOI:** 10.1038/s41598-020-75141-2

**Published:** 2020-10-22

**Authors:** Lan-Lan Xiao, Fen Zhang, Ya-Lei Zhao, Ling-Jian Zhang, Zhong-Yang Xie, Kai-Zhou Huang, Xiao-Xi Ouyang, Xiao-Xin Wu, Xiao-Wei Xu, Lan-Juan Li

**Affiliations:** 1 State Key Laboratory for Diagnosis and Treatment of Infectious Diseases, National Clinical Research Center for Infectious Diseases, Collaborative Innovation Center for Diagnosis and Treatment of Infectious Diseases, The First Affiliated Hospital, College of Medicine, Zhejiang University, Hangzhou, China; 2 Shantou Central Hospital, Affiliated Shantou Hospital of Sun Yat-Sen University, Guangdong, China

**Keywords:** Biomarkers, Diseases, Gastroenterology

## Abstract

Increased oxidative stress levels play a key role in idiosyncratic drug-induced liver injury (DILI) pathogenesis. To investigated whether advanced oxidation protein products (AOPPs) and ischaemia-modified albumin (IMA) can be used to monitor oxidative stress in DILI patients and to assess disease severity. We performed spectrophotometric assays to assess AOPPs and IMA in 68 DILI patients with severity grade 0–2 (non-severe group), 60 with severity grade 3–5 (severe group), and 38 healthy controls. The results showed that baseline AOPPs and IMA serum levels and AOPPs/albumin and IMA/albumin ratios were significantly higher in DILI patients than in healthy controls. Besides, in comparison to the non-severe group, the severe group showed higher baseline AOPPs and IMA serum levels and AOPPs/albumin and IMA/albumin ratios. AOPPs and IMA serum levels and AOPPs/albumin and IMA/albumin ratios decreased after treatment in both patient groups. Combining the correlation analysis and areas under the receiver operating curve (AUROCs) analysis results, that IMA outperformed to be one is the most reliable marker to assess disease severity of DILI. Our findings indicated that AOPPs and IMA can serve as key biomarkers for monitoring oxidative stress levels in DILI patients and can indicate disease severity. The IMA outperformed to be one of the most reliable oxidative stress biomarkers to assess disease severity of DILI.

## Introduction

Idiosyncratic drug-induced liver injury (DILI) is a rare clinical disorder and can lead to jaundice, liver failure or even death^1^. Its annual morbidity in developed nations approximately ranges between 1/100,000 and 20/100,000^2,3^. In China, the acute DILI accounts for approximately 20% of ALF-related hospitalizations^4^; however, a reliable incidence rate for DILI in the general population remains unknown. People who develop hepatocellular DILI with jaundice show a mortality rate of at least 10%, and patients with DILI who eventually develop ALF show only a 25% chance of spontaneous recovery^5^. Considering the wide range of presentations and array of drugs used in clinical practice and due to the lack of specific biomarkers, the diagnosing DILI is still a particularly difficult process; in fact, DILI remains one of the most challenging liver disorders faced by hepatologists^6^.

The pathogenesis of DILI is not well characterized. The probable pathogenesis of idiosyncratic DILI involves the inadvertent generation of a reactive metabolite or drug–protein complex that can directly or indirectly mediate damage to intracellular proteins and/or organelles, consequently inducing ‘danger’ signals (oxidative stress, mitochondrial damage, endoplasmic reticulum stress and bile salt export pump inhibition)^6^. Oxidative stress reportedly plays a key role in DILI, which is known to affect cell membranes, proteins and DNA and can lead to apoptosis and cell death, ultimately causing liver dysfunction. The liver is vulnerable to oxidative stress, and sustained oxidative stress has been suggested to play a pivotal role in the initiation and progression of liver diseases^7,8^.

Witko-Sarsat et al. in 1996 proposed the measurement of advanced oxidation protein products (AOPPs) to reliably estimate the degree of oxidant-mediated protein damage in uremic patients^9^. Since then, AOPPs have been used as an oxidative stress biomarker^9,10^ and inflammation mediators^11,12^. They evidently play a critical role in the pathological process of diverse diseases and their complications, particularly in chronic kidney diseases^13^. In the past decade, advances have been made to understand the role of AOPPs in various liver diseases^14^; however, there is little clinical evidence regarding whether a potential link exists between AOPPs and acute liver injury, specifically in case of DILI.

Ischaemia-modified albumin (IMA) is a known myocardial infarction biomarker^15^; nevertheless, IMA levels may also increase in conditions of non-cardiac ischaemia, such as liver cirrhosis and metabolic syndrome^16,17^. The albumin (ALB) molecule in the plasma of diabetic patients is modified in chronic hypoxia conditions, mainly provoked by hyperglycaemia and oxidative stress^18^. Several studies have demonstrated that reactive oxygen species (ROS) can generate the highly reactive hydroxyl radical, causing a conformational change to the N-terminus of the ALB moiety and eventually resulting in IMA production^19^. IMA is a novel oxidative stress biomarker that has been shown to be elevated in chronic liver diseases^20^. Despite this, to date, the relationship between IMA and DILI development remains unexplained.

In this study, we focused on AOPPs and IMA serum levels and determined AOPPs/ALB and IMA/ALB ratios in patients with DILI during treatment. Moreover, we explored the association between oxidative stress biomarkers and severity and prognosis of DILI.

## Results

### Characteristics of enrolled subjects

Between January 2018 and October 2019, 167 inpatients were screened. Only 128 patients with ‘highly probable’ or ‘probable’ causality and 38 healthy controls (HCs) were eventually enrolled. Of the 128 patients with DILI, 68 were included in the non-severe group and 60 in the severe group. Almost all patients recovered after treatment; one patient in the severe group died within 90 days. 25 (19.5%) patients were developed to chronic DILI, 14 (20.6%) in non-severe group, and 11 (18.3%) in severe group. The most common pattern of liver injury was hepatocellular (observed in 102 [79.7%] and 48 [80.0%] patients in the non-severe and severe groups, respectively). In comparison to patients in the non-severe group, those in the severe group tended to be clinically jaundiced (P < 0.001), were less likely to have diabetes and tended to have higher alanine aminotransferase (ALT) and total bilirubin (TBIL) levels (P = 0.022 and P < 0.001, respectively). Herbal or dietary supplements were the most common causative agents, accounting for 98 (76.6%) cases; other causative agents included antimicrobials [7 (5.5%) cases] and cardiovascular drugs [4 (3.1%) cases]. The characteristics of all participants are listed in Table 1.

**Table 1:** Demographic, clinical, and laboratory parameters of the subjects.

	DILI			Healthy control (n = 38)	P
	All patients (n = 128)	Non-severe group (n = 68)	Severe group (n = 60)		
Age (year, mean [SD])	50.61 (14.8)	50.2 (15.6)	51.1 (14.1)	44.0 (13.2)	0.792
Female (%)	89 (69.5)	49 (72.1)	40 (66.7)	21 (55.3)	0.508
Alcohol use	20 (15.6)	8 (11.8)	12 (20.0)	–	0.200
Preexiting liver disease	28 (21.9)	16 (23.5)	12 (20.0)	–	0.630
Hypertension	22 (17.2)	12 (17.6)	10 (16.7)	–	0.883
Diabetes mellitus	20 (15.6)	16 (23.5)	4 (6.7)	–	0.009
Jaundice	52 (40.6)	12 (17.6)	40 (66.7)	–	< 0.001
Causative drugs-HDS	98 (76.6)	46 (67.6)	52 (86.7)	–	0.011
**Latency**					0.258
< 5 days	9 (7.0)	5 (7.4)	4 (6.7)	–	
5–90 days	111 (86.7)	61 (89.7)	50 (83.3)	–	
> 90 days	8 (6.3)	2 (2.9)	6 (10.0)	–	
**RUCAM**					0.649
Highly probable (> 8)	74	39	35	–	
Probable (6–8)	46	21	25	–	
**Pattern of liver injury (%)**					0.453
Hepatocellular	102 (79.7)	54 (79.4)	48 (80.0)	–	
Cholestatic	8 (6.3)	4 (5.9)	4 (6.7)	–	
Mixed	18 (14.1)	10 (14.7)	8 (13.3)	–	
Death by 90th day	1 (0.8)	0	1 (3.9)	–	0.285
**Liver biochemistries at admission**					
Chronic DILI	25 (19.5)	14 (20.6)	11 (18.3)	–	0.748
ALT (U/L)	665.02 (486.1)	535.12 (342.9)	812.2 (580.8)	20.6 (9.9)	0.022
AST (U/L)	382.9 (311.1)	272.6 (169.8)	507.9 (383.4)	19.7 (5.6)	0.004
ALP (U/L)	68.9 (15.7)	158.4 (75.2)	183.3 (90.7)	68.9 (15.7)	0.235
TBIL (mg/dL)	5.8 (5.9)	1.8 (1.3)	10.4 (5.7)	0.7 (0.3)	< 0.001
GGT (U/L)	206.7 (183.2)	186.5 (166.3)	228.2 (200.2)	25.1 (15.7)	0.379
ALB (g/L)	38.9 (4.2)	39.9 (3.7)	37.7 (4.4)	46.5 (3.4)	0.036
INR	1.0 (0.2)	1.0 (0.1)	1.0 (0.2)	–	0.079
AOPPs (μmol/L)	176.8 (92.6)	124.8 (61.8)	235.7 (86.5)	40.1 (14.2)	< 0.001
AOPPs/Albumin (μmol/g)	5.1 (3.1)	3.1 (1.2)	7.4 (2.9)	0.8 (0.3)	< 0.001
IMA (ABSU)	1.3 (0.8)	0.7 (0.3)	1.8 (0.7)	0.3 (0.1)	< 0.001
IMA/Albumin (ABSU*dL/g)	0.3 (0.2)	0.2 (0.1)	0.5 (0.2)	0.07 (0.02)	< 0.001
**Liver biochemistries on discharge**					
ALT (U/L)	205.4 (161.1)	193.4 (123.8)	219.8 (198.4)	–	0.518
AST (U/L)	96.6 (71.4)	85.3 (63.7)	109.5 (78.2)	–	0.184
ALP (U/L)	139.0 (62.1)	145.5 (74.3)	131.2 (43.0)	–	0.364
TBIL (mg/dL)	3.1 (3.2)	1.4 (1.4)	5.1 (3.7)	–	< 0.001
GGT (U/L)	172.4 (185.8)	155.1 (147.1)	193.3 (224.8)	–	0.418
ALB (g/L)	37.09 (4.0)	38.0 (3.7)	36.0 (4.2)	–	0.518
INR	1.0 (0.1)	0.9 (0.1)	1.0 (0.2)	–	0.014
AOPPs (μmol/L)	96.6 (52.2)	77.8 (30.3)	118.0 (62.8)	–	< 0.001
AOPPs/Albumin (μmol/g)	2.7 (2.0)	1.9 (0.6)	3.5 (2.5)	–	< 0.001
IMA (ABSU)	0.8 (0.5)	0.6 (0.2)	1.1 (0.5)	–	< 0.001
IMA/Albumin (ABSU*dL/g)	0.2 (0.1)	0.1 (0.06)	0.3 (0.1)	–	< 0.001

Data are mean ± SD, or number (percentage). P-values for comparisons between non-severe group and severe group.

*DILI* drug-induced liver injury, *SD* standard deviation, *HDS* herbal or dietary supplements, *RUCAM* Roussel Uclaf causality assessment method, *ALT* alanine aminotransferase, *AST* aspartate aminotransferase, *ALP* alkaline phosphatase, *TBIL* total bilirubin, *GGT* γ-glutamyl transpeptidase, *ALB* albumin, *INR* international normalized ratio, *AOPPs* advanced oxidation protein products, *IMA* ischemia-modified albumin.

### AOPPs serum levels and AOPPs/ALB ratio

**Figure 1:**
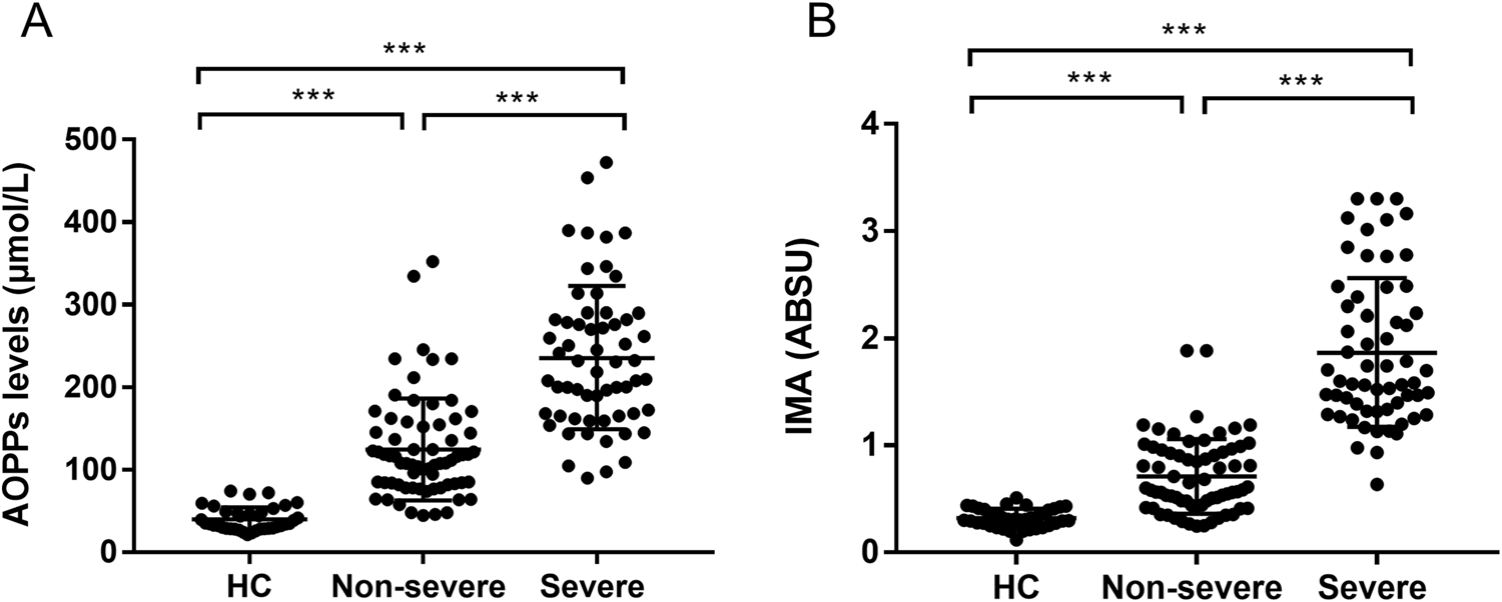
Baseline serum levels of advanced oxidation protein products (AOPPs) and ischaemia-modified albumin (IMA) in patients with drug-induced liver injury and healthy controls (HCs). (**A**) AOPPs levels. (**B**) IMA levels. ***P < 0.001. *ABSU* absorbance units.

**Figure 2:**
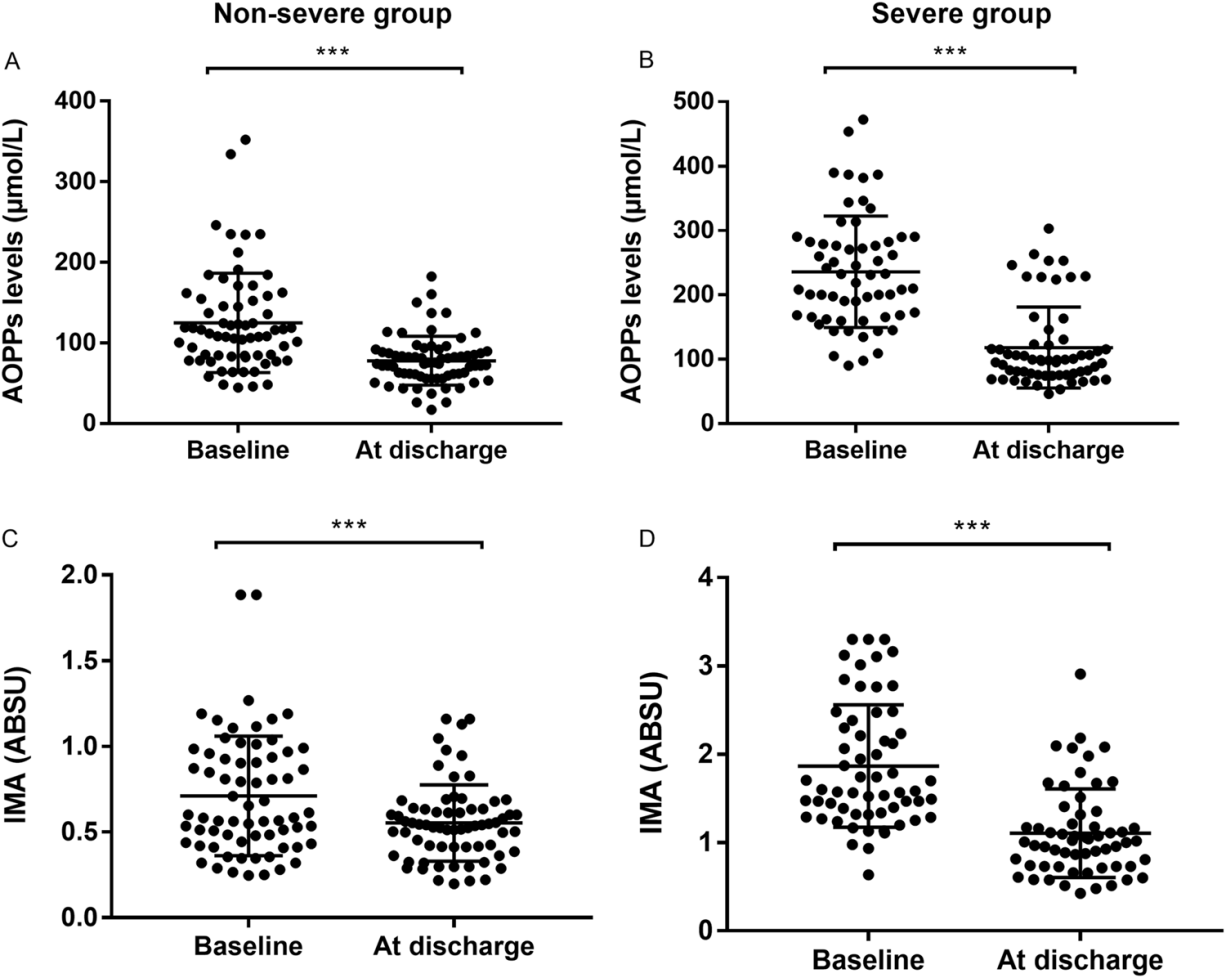
Serum levels of advanced oxidation protein products (AOPPs) and ischaemia-modified albumin (IMA) in patients with drug-induced liver injury before and after treatment. (**A**) AOPPs levels in the non-severe group. (**B**) AOPPs levels in the severe group. (**C**) IMA levels in the non-severe group. (**D**) IMA levels in the severe group. ***P < 0.001. *ABSU* absorbance units.

**Figure 3:**
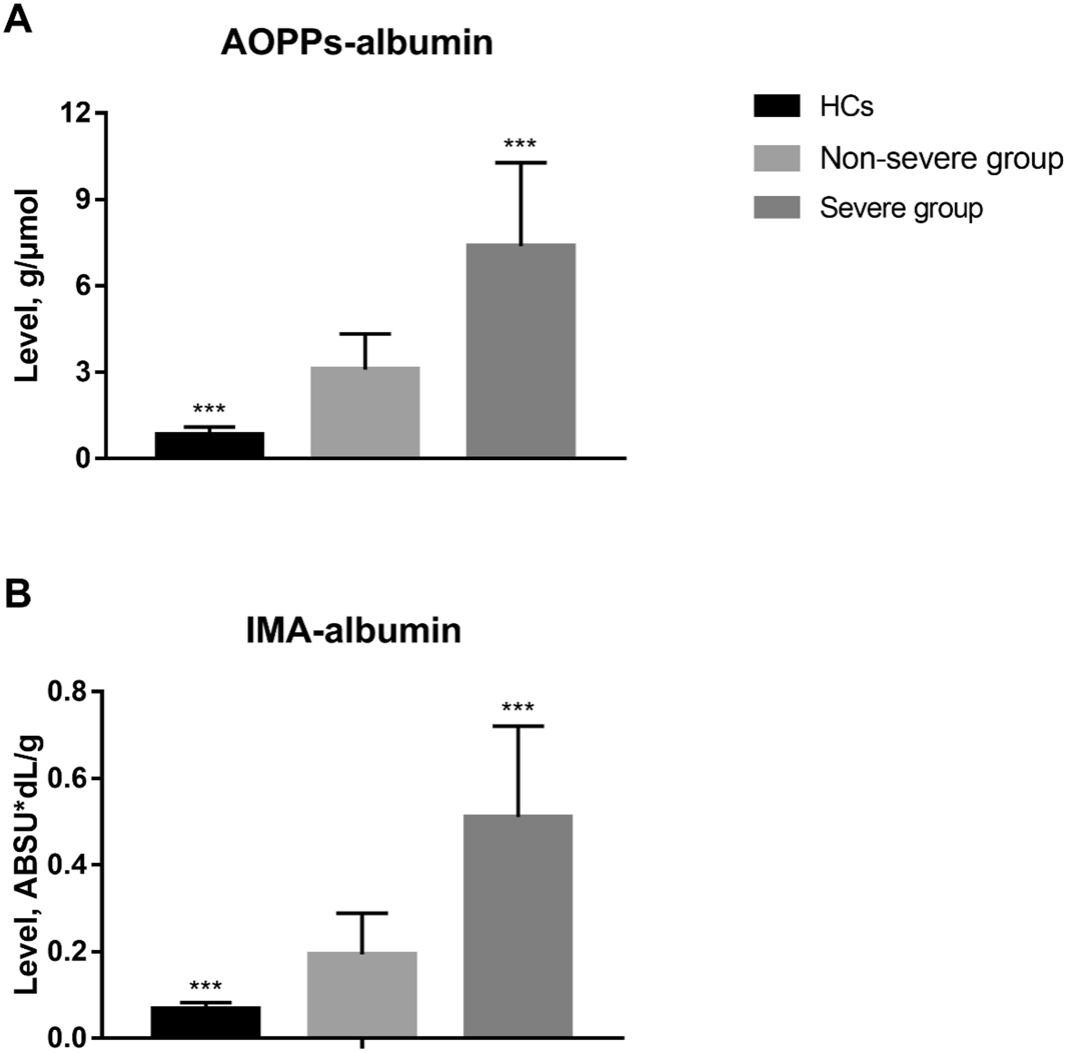
Baseline advanced oxidation protein products (AOPPs)/albumin and ischaemia-modified albumin (IMA)/albumin ratios in patients with drug-induced liver injury and healthy controls (HCs). (A) AOPPs/albumin ratio. (**B**) IMA/albumin ratio. ***P < 0.001. *ABSU* absorbance units.

**Figure 4:**
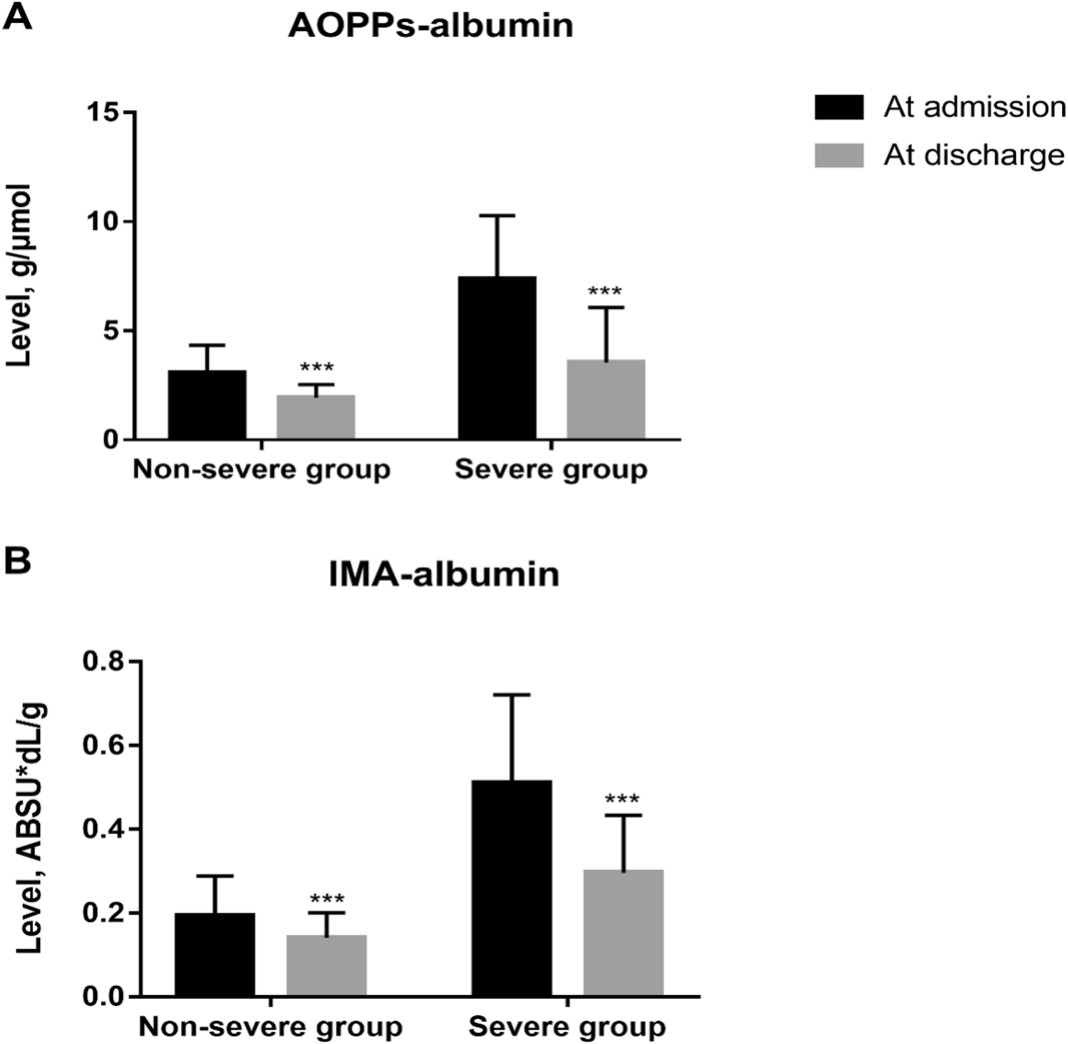
Advanced oxidation protein products (AOPPs)/albumin and ischaemia-modified albumin (IMA)/albumin ratios in patients with drug-induced liver injury before and after treatment. (**A**) AOPPs/albumin ratio in the non-severe and severe groups before and after treatment. (**B**) IMA/albumin ratio in the non-severe and severe groups before and after treatment. ***P < 0.001. *ABSU* absorbance units.

At admission, patients with DILI showed significantly higher AOPPs serum levels (176.8 ± 92.6 μmol/L) and AOPPs/ALB ratio (5.1 ± 3.1 μmol/g) as compared to those shown by HCs (40.1 ± 14.2 μmol/L and 0.8 ± 0.3 μmol/g, respectively, P < 0.001; Table 1). In comparison to the non-severe group, baseline AOPPs serum levels and AOPPs/ALB ratio was noticeably higher in the severe group (124.8 ± 61.8 μmol/L vs. 235.7 ± 86.5 μmol/L and 3.1 ± 1.2 μmol/g vs. 7.4 ± 2.9 μmol/g, respectively, P < 0.001; Figs. 1A, 3A). After treatment, AOPPs serum levels and AOPPs/ALB ratio (at discharge) significantly decreased both in the non-severe and severe groups (P < 0.001, respectively; Figs. 2A,B, 4A).

### IMA serum levels and IMA/ALB ratio

At admission, patients with DILI showed significantly higher IMA serum levels [1.3 ± 0.8 absorbance units (ABSU)] and IMA/ALB ratio (0.3 ± 0.2 ABSU*dL/g) as compared to those shown by HCs (0.3 ± 0.1 ABSU and 0.07 ± 0.02 ABSU*dL/g, respectively, P < 0.001; Table 1). Further, patients in the non-severe group showed lower IMA serum levels and IMA/ALB ratio than those in the severe group (0.7 ± 0.3 ABSU vs. 1.8 ± 0.7 ABSU and 0.2 ± 0.1 ABSU*dL/g vs. 0.5 ± 0.2 ABSU*dL/g, respectively, P < 0.001; Figs. 1B, 3B). After treatment, IMA serum levels and IMA/ALB ratio decreased both in the non-severe group and severe groups (P < 0.001, respectively; Figs. 2C,D, 4B).

### The performances of oxidative stress biomarkers in assessment of severity for patients with DILI

**Figure 5:**
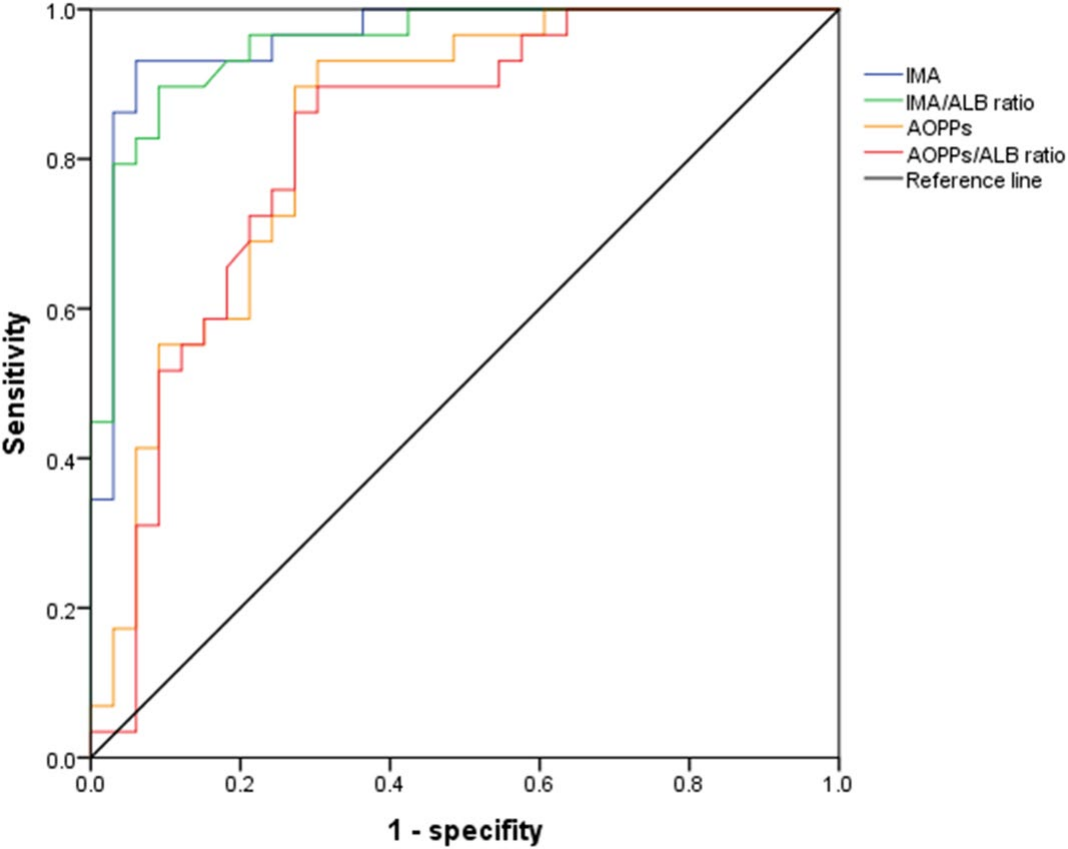
The areas under the receiver operating curve for oxidative stress biomarkers to predict the severe DILI.

Using the baseline data of 128 patients with DILI, we analysed the correlation between oxidative stress biomarkers and clinical parameters (Table 2). Interestingly, that AOPPs, AOPPs/ALB ratio, IMA, IMA/ALB ratio were all positively correlated with alkaline phosphatase (ALP; r = 0.306, 0.276, 0.276 and 0.315; P = 0.016, 0.031, 0.031 and 0.013, respectively) and TBIL (r = 0.305, 0.360, 0.809 and 0.779; P = 0.017, 0.004, < 0.001 and < 0.001, respectively). We also found that all oxidative stress biomarkers have strong relationship with the severity, however, the IMA had the higher R value than AOPPs, AOPPs/ALB ratio, and IMA/ALB ratio (Table 2). To compare the predictive value of different oxidative stress biomarkers for severe DILI, then the areas under the receiver operating curve (AUROCs) were calculated in this study. The AUROCs of AOPPs, IMA, AOPPs/ALB ratio, IMA/ALB ratio for diagnosis of severe DILI were 0.839, 0.959, 0.821 and 0.954, respectively (Fig. 5). Combining the correlation analysis and AUROCs analysis results, that IMA outperformed, or was at least comparable with, any one of AOPPs, AOPPs/ALB ratio, IMA/ALB ratio, one of the most reliable markers to assess disease severity of DILI.

**Table 2:** Correlations between oxidative stress biomarkers and other parameters in patients with drug-induced liver injury.

Characteristics	AOPPs	AOPPs/albumin ratio	IMA	IMA/albumin ratio
ALT	0.263 (0.146)	0.284 (0.139)	0.807 (0.032)	0.736 (0.044)
AST	0.627 (0.064)	0.701 (0.05)	0.525 (0.083)	0.63 (0.063)
ALP	0.016 (0.306)	0.031 (0.276)	0.031 (0.276)	0.013 (0.315)
TBIL	0.017 (0.305)	0.004 (0.360)	< 0.001 (0.809)	< 0.001(0.779)
TBA	0.374 (0.116)	0.429 (0.103)	0.01 (0.330)	0.02 (0.298)
GGT	0.062 (0.241)	0.235 (0.154)	0.009 (0.334)	0.048 (0.254)
Severity	< 0.001 (0.489)	< 0.001 (0.511)	< 0.001 (0.726)	< 0.001(0.695)
Chronic DILI	0.736 (0.044)	0.491 (0.089)	0.886 (− 0.019)	0.856 (0.024)
Hospitalization time	0.21 (0.12)	0.202 (0.116)	0.323 (0.01)	0.294 (0.02)
AOPPs	–	–	< 0.001 (0.509)	< 0.001(0.481)
AOPPs/Albumin ratio	–	–	< 0.001 (0.569)	< 0.001(0.582)

Data are P value (r value).

*AOPPs* advanced oxidation protein products, *IMA* ischemia-modified albumin, *ALT* alanine aminotransferase, *AST* aspartate aminotransferase, *ALP* alkaline phosphatase, *TBIL* total bilirubin, *TBA* total bile acid, *GGT* γ-glutamyl transpeptidase.

We found that AOPPs had a positive correlation with IMA. Here, we conducted a multivariate logistic regression model with AOPPs and IMA to diagnosis the severe DILI. It came out that both AOPPs and IMA were both risk factors for severe DILI (Supplementary Material Table 3). Accordingly, we hypothesized that patient with higher AOPPs and higher IMA serum is more likely to suffer from severe DILI. While patient with lower AOPPs and lower IMA serum level is more likely to suffer from mild DILI.

### Could oxidative stress biomarkers predict the prognosis of DILI?

In our study, 128 patients were followed up at least 6 months. Meanwhile, we try to analyze the correlation between baseline oxidative stress biomarkers (AOPPs and IMA levels or AOPPs/albumin or IMA/albumin ratio) and chronic DILI. Unfortunately, there were no significant correlation between oxidative stress biomarkers (AOPPs and IMA levels or AOPPs/albumin or IMA/albumin ratio) and chronic DILI (Table 2). Therefore, these oxidative stress biomarkers could not predict the chronic DILI.

Though each person left hospital with a different degree of illness that we still try to analyze the correlation between baseline oxidative stress biomarkers and hospitalization time. We found that IMA and IMA/albumin ratio had a positive correlation with hospitalization time (r = 0.323 and 0.294; P = 0.01 and 0.02, respectively). While we did not find any significant correlation between AOPPs and AOPPs/albumin and hospitalization time (Table 2). Patient with higher serum IMA and IMA/albumin ratio might along with longer hospitalization time. However, above results could not demonstrate that the oxidative stress biomarkers are prognostic biomarkers of DILI.

## Discussion

Herein we investigated the role of oxidative stress biomarkers (AOPPs, IMA, AOPPs/ALB ratio, IMA/ALB ratio) in patients with DILI. We found that in comparison with HCs, patients with DILI showed higher levels of circulating serum oxidative stress biomarkers. Patients in the severe group showed significantly higher AOPP and IMA serum levels as well as AOPP/ALB and IMA/ALB ratios. The levels of oxidative stress biomarkers showed an obvious decrease after treatment. According to the correlation analysis and AUROCs analysis results, that IMA outperformed to be one is the most reliable marker to assess disease severity of DILI.

Majority of patients who experience DILI will fully recover clinically and biochemically. Although part of DILI was implicated with acute liver failure, the mortality was low^6^. In our study, the mortality rate for patients with DILI was 0.8% (1/128). Therefore, the mortality is not a reasonable indicator to assess the prognosis. Evidence showed that a significant number of patients who suffer from DILI will progress to chronic DILI, which has been defined by Drug Induced Liver Injury Network (DILIN) as continued injury 6 months after the initial diagnosis^21^. We conducted the correlation analysis between baseline oxidative stress biomarkers and chronic DILI, however, no significant results were found. We are very sorry to find that oxidative stress biomarkers might not predict the prognosis of DILI.

**Figure 6:**
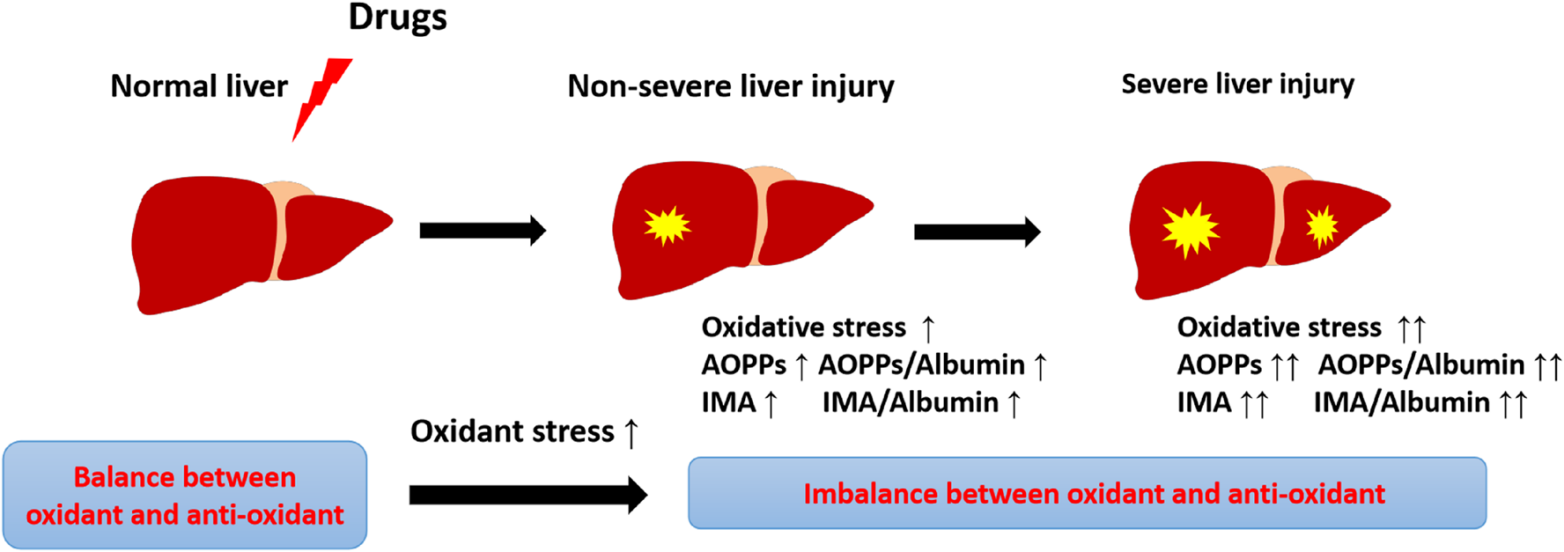
Generation of advanced oxidation protein products (AOPPs) and ischaemia-modified albumin (IMA) in drug-induced liver injury. Upon exposure to drugs, the liver experiences oxidative stress. A series of pathophysiological events occur, particularly oxidative stress, mitochondrial damage, endoplasmic reticulum stress and bile salt export pump inhibition. Finally, AOPPs and IMA are generated.

Oxidative stress is central to the pathogenesis of many liver diseases^7^. There exists a balance between oxidants and antioxidants in the healthy liver. In mammals, a sophisticated antioxidant system is present to maintain redox homeostasis in the liver^22^. However, when the liver is exposed to exogenous and/or endogenous factors such as drugs, viruses or alcohol, the oxidant–antioxidant balance is disturbed by ‘oxidative stress’, resulting in the generation of AOPPs and IMA (Fig. 6).

The pathogenesis of DILI potentially involves three mechanisms that are responsible for the majority of cases with intrinsic and idiosyncratic DILI—mitochondrial dysfunction, oxidative stress and alterations in bile acid homeostasis^23–25^. Oxidative stress can be induced by ROS, which are produced during normal metabolism and involved in cell signalling and homeostasis. However, some DILI-causing agents are known to increase ROS accumulation through various mechanisms. Consequently, the elimination of excessive ROS can result in oxidative stress, which causes damage to key cellular constituents and even cell death^23^.

The role of AOPPs is being investigated in an increasing number of animal experiments and clinical liver diseases^14^. Sun et al. reported that plasma AOPPs levels were higher in rats with acetaminophen-induced liver failure as compared with those in normal rats. They also revealed that plasma AOPPs levels were correlated with the severity of acetaminophen-induced liver injury^26^. Further, an increase in plasma or liver AOPPs was reported in an experimental liver injury model induced by the widely used hepatotoxic carbon tetrachloride and in the liver tissues from rats with liver damage induced by bile duct ligation. Several studies have reported the clinical relevance of AOPPs in liver diseases, such as chronic hepatitis C, alcohol-induced liver injury, non-alcoholic fatty liver disease, non-alcoholic steatohepatitis, liver cirrhosis, acute-on-chronic liver failure and hepatocellular carcinoma^14^. However, very little information exists on the role of AOPPs in DILI, and only a few biomarkers are available to assess oxidative stress levels.

AOPPs can be generated upon the exposure of serum ALB to hypochlorous acid in vitro; plasma AOPPs are mainly carried by ALB in vivo^27^. A study reported high plasma AOPPs/ALB ratio in cirrhotic patients with chronic hepatitis C^28^. In this study, patients with DILI had high AOPPs serum levels and AOPPs/ALB ratio—we thus propose that these can serve as oxidative stress biomarkers and can be used to assess DILI severity. AOPPs measurements reflect free radical generation and protein oxidation extent^9^. In vitro, AOPPs are known to upregulate transforming growth factor-β receptor I in hepatocytes and cause oxidative stress^29^.

IMA is gaining popularity as a biomarker; it has been used to evaluate the overall level of ischaemic damage and oxidative stress^20^. Kumar et al. revealed that IMA can serve as an oxidative stress biomarker to assess disease severity and prognosis in patients with chronic liver disease^17^. Moreover, IMA levels have been found to be elevated along with other biomarkers of oxidative stress, such as total oxidant status levels and oxidative stress index^30^. Oxidative stress induces a site-specific modification at the N-terminus of the ALB molecule; such an alteration consequently affects the ability of the N-terminus of ALB to bind metals. This modified form of ALB is known as IMA^19^. This process indicates that IMA levels tend to increase with a decrease in ALB levels. In addition, a study found a negative correlation between IMA and ALB levels in patients with chronic liver disease^20^. In this study, even we found a strong negative correlation between ALB and IMA levels (P = 0.001). Thus, we believe that while there may be other unrecognized forms of ALB in patients with DILI, IMA generation specifically plays a significant role in decreasing ALB levels. The serum levels of AOPPs as well as those of IMA have been reported to be higher in patients with diabetes^20^. However, AOPPs and IMA serum levels in addition to AOPPs/ALB and IMA/ALB ratios showed no significant difference between DILI patients with diabetes and without diabetes. The mechanism underlying this phenomenon remains unclear.

The absence of specific diagnostic biomarkers for DILI makes differential diagnoses strongly dependent on the judicious interpretation of serum liver biochemistry and other routine laboratory and imaging test results. ALT, ALP and TBIL serum levels remain the mainstay for detecting and classifying liver damage in suspected DILI^31^. Elevated ALT serum levels along with a concomitant increase in TBIL levels can potentially serve as a reliable biomarker of liver injury in DILI^32^. Oxidative stress induced by ROS is believed to be crucial in the pathogenesis of acute and chronic liver diseases, regardless of the aetiology^7^. We observed an evident distinction in the levels of oxidative stress biomarkers between the severe and non-severe groups; further, the serum levels of these biomarkers correlated with liver biochemistry (ALP, TBIL, GGT, TBA and ALB). Thus, our data suggest that these oxidative stress biomarkers can be used to gain insights into DILI severity and outcomes. In addition, they may improve the speed and/or accuracy of diagnosing DILI.

The biggest limitation in our study is the small sample size, which potentially resulted in weaker associations among some of the parameters. More extensive confirmatory studies should be performed to better estimate the association between AOPPs, IMA and patients with DILI.

In conclusion, we report that patients with DILI have high oxidative stress levels and that effective treatment can tackle this condition. We propose that AOPPs and IMA serum levels in addition to AOPPs/ALB and IMA/ALB ratios are suitable to assess and monitor oxidative stress levels in patients with DILI and to determine disease severity. More importantly, these oxidative stress biomarkers accompanied by serum liver biochemistry can indicate the development of DILI. However, further large-scale studies need to be conducted to validate our findings.

## Methods

### Patients

This single-centred study was conducted between January 2018 and October 2019 at the First Affiliated Hospital, Zhejiang University School of Medicine. Hospitalized patients diagnosed with idiosyncratic DILI were enrolled. Relevant clinical, biochemical, serological and histological data were collected, and, if not already performed, correlation tests were carried out for identifying suspected DILI, including hepatitis A, B, C and E, CMV, EBV, HSV and autoimmune hepatitis. All patients received integrative treatment after discharge. First, patients were required to promptly discontinue suspected liver injury drugs and try to avoid the use of suspected or similar drugs. A low-salt and low-fat diet was highly recommended; N-Acetyl-L-cysteine, reducing glutathione, ademetionine, compound glycyrrhizin tablets or ursodeoxycholic acid were used for improving liver function. Liver transplantation was considered for DILI patients with severe hepatic encephalopathy and coagulation disorders, or decompensated liver cirrhosis^33^. All patients were followed up at least 6 months (by telephone and outpatient record enquiry).

Blood samples were obtained both on the day of admission and at discharge, and subsequently centrifuged at 4 °C and 3500 rpm for 10 min; the obtained serum samples were stored at − 80 °C. This study was conducted in compliance with the ethical principles of the Declaration of Helsinki and approved by the Ethics Committee of the First Affiliated Hospital, Zhejiang University School of Medicine. All patients who participated in the study provided signed informed consent.

### Inclusion and exclusion criteria

According to the DILIN study^34^, patients with a medication history and hepatic biochemical abnormality who met one or more of the following criteria were included: (1) jaundice (serum TBIL ≥ 2.5 mg/dL) or coagulopathy [international normalised ratio (INR) > 1.5] with elevated ALT or aspartate aminotransferase (AST) or ALP levels and (2) in the absence of jaundice or coagulopathy, elevated ALT or AST levels > 5 times the upper limit of normal (ULN) or ALP levels > 2 times the ULN.

The exclusion criteria were as follows: (1) age < 18 years or > 80 years, (2) liver injury caused by hepatitis viruses, autoimmune liver diseases, metabolic liver diseases or liver cancer, (3) human immunodeficiency virus infection, (4) liver or bone marrow transplantation prior to enrolment and (5) presence of a severe comorbidity that could affect treatment.

### Severity assessment

The degree of severity was defined according to the DILIN study^34^ as follows: mild (1 +), elevated serum enzyme (ALT and/or ALP) levels in the absence of jaundice (TBIL < 2.5 mg/dL); moderate (2 +), elevated serum enzyme levels along with jaundice (TBIL ≥ 2.5 mg/dL) or coagulopathy (INR > 1.5) but without the need for hospitalization; moderately severe (3 +), elevated serum enzyme levels along with jaundice or coagulopathy and with the need for hospitalization; severe (4 +), jaundice and signs of hepatic or other organ failure (i.e. renal or pulmonary) and fatal (5 +), death or liver transplantation from a DILI event.

### Clinical patterns of liver injury and causality assessment

The pattern of liver injury was assessed based on the R value [(ALT value/ALT UNL) / (ALP value/ALP UNL)]. By convention, hepatocellular DILI is defined as R ≥ 5, cholestatic DILI as R ≤ 2, and mixed DILI as R > 2 and < 5^35^. The R ratio applied to each case was calculated based upon the values at admission. The Roussel Uclaf Causality Assessment Method was used to evaluate causality of relationships identified, if any. Causality was assessed as either highly probable (> 8), probable (6–8), possible (3–5), unlikely (1 or 2) or excluded (0)^35,36^.

### Measurement of serum AOPPs and IMA

AOPP determination was based on a spectrophotometry-based method reported by Witko-Sarsat et al*.*^9^. We measured AOPP serum levels using an OxiSelect AOPP Assay Kit (Cell Biolabs, CA, USA). IMA was measured using a colorimetric assay, as previously reported by Bar-Or et al.^37^ Briefly, 100 μL serum was added to 25 μL of 0.1% (w/v) cobalt chloridine water solution (Sigma, CoCl_2_ × 6H_2_O), gently mixed, and incubated for 10 min to allow sufficient cobalt–ALB binding. Subsequently, 25 μL dithiothreitol (DTT) (Sigma, 1.5 mg/mL H_2_O) was added as the colorizing agent, followed by incubation for 2 min. Finally, 150 μL of 0.9% NaCl was added to stop the reaction. Absorbance was measured at 492 nm using a spectrophotometer (Epoch 2 Microplate Spectrophotometer); colour development of the sample with DTT was compared to that of a sample without DTT (values reported as ABSU).

### Statistical analysis

Descriptive statistics are expressed as means with standard deviation or number with percentage. Categorical data were compared using the chi-square or Fisher’s exact test. Continuous variables were compared using the Wilcoxon/Kruskal–Wallis test. The correlation analysis was performed to identify relationship between oxidative stress biomarkers and clinical parameters. To compare the predictive value of different oxidative stress biomarkers, AUROCs were calculated. Statistical analyses were performed using IBM SPSS v24.0 for Windows (IBM Corp., Armonk, NY, USA). P < 0.05 indicated statistical significance.

## Supplementary information

Supplementary Information.

## Data Availability

Datasets and original images are available from the corresponding author on request.
