# Supplementary material for: Using advanced oxidation protein products and ischaemia-modified albumin to monitor oxidative stress levels in patients with drug-induced liver injury
Source: Sci Rep. 2020 Oct 22;10:18128. doi: 10.1038/s41598-020-75141-2 (PMC7582878; doi:10.1038/s41598-020-75141-2)
Supplement: Supplementary file 1 — Supplementary Information. [file 41598_2020_75141_MOESM1_ESM.doc]

**Using advanced oxidation protein products and ischaemia-modified albumin to monitor oxidative stress levels in** **patients with** **drug-induced liver injury**

Lan-Lan Xiao^1^, Fen Zhang^1^, Ya-Lei Zhao^1^, Ling-Jian Zhang^1^, Zhong-Yang Xie^1^, Kai-Zhou Huang^2^, Xiao-Xi Ouyang^1^, Xiao-Xin Wu^1^, Xiao-Wei Xu^1^*, Lan-Juan Li^1^*

**Supplementary material**

Supplementary material Table 1. The use of N-Acetyl-L-cysteine and reducing glutathione in each group

|  | Non-severe group (n = 68) | Severe group (n = 60) | p |
| --- | --- | --- | --- |
| N-Acetyl-L-cysteine | 41 (60.3%) | 44 (73.3%) | 0.119 |
| Reducing glutathione | 40 (58.8%) | 37 (61.7%) | 0.743 |
| Neither N-Acetyl-L-cysteine nor reducing glutathione | 6 (8.8%) | 3 (5.0%) | 0.398 |

Supplementary material Table 2. The coefficient of variation (CV%) of intraassay and interassay for the IMA and AOPP

|  | IMA | | | | | AOPPs | | | | |
| --- | --- | --- | --- | --- | --- | --- | --- | --- | --- | --- |
|  | intraassay | | | | CV% interassay | intraassay | | | | CV% interassay |
|  | CV1 | CV2 | CV3 | CV4 |  | CV1 | CV2 | CV3 | CV4 |  |
| Non-severe group |  |  |  |  |  |  |  |  |  |  |
| At admission | 30.6% | 32.1% | 33.4% | 28.5% | 9.6% | 39.6% | 32.4% | 35.1% | 36.3% | 14.1% |
| At discharge | 27.9% | 29.5% | 30.1% | 26.4% | 14.5% | 28.8% | 35.3% | 27.8% | 30.5% | 13.3% |
| Severe group |  |  |  |  |  |  |  |  |  |  |
| At admission | 31.3% | 30.8% | 28.9% | 31.6% | 9.9% | 36.0% | 31.3% | 29.3% | 27.8% | 5.9% |
| At discharge | 33.4% | 32.6% | 26.8% | 30.4% | 0.8% | 24.2% | 23.7% | 22.6% | 25.5% | 7.3% |
| Healthy controls | 28.7% | 27.2% | 30.2% | 26.1% | 4.2% | 37.8% | 36.9% | 30.1% | 34.6% | 30.8% |

AOPPs, advanced oxidation protein products; IMA, ischemia-modified albumin.

Supplementary material Table 3. Multivariate logistic regression model with AOPP and IMA investigating severe DILI.

| Variable | B | Reference | OR (CI 95 %) | *p* value |
| --- | --- | --- | --- | --- |
| IMA | 4.667 | Per unit increase | 106.329 (2.883-3922.245) | 0.011 |
| AOPP | 0.023 | Per unit increase | 1.023 (1.004-1.042) | 0.017 |
